# Prediction of Early Childhood Caries Based on Single Nucleotide Polymorphisms Using Neural Networks

**DOI:** 10.3390/genes12040462

**Published:** 2021-03-24

**Authors:** Katarzyna Zaorska, Tomasz Szczapa, Maria Borysewicz-Lewicka, Michał Nowicki, Karolina Gerreth

**Affiliations:** 1 Department of Histology and Embryology, Poznan University of Medical Sciences, 6 Swiecickiego Street, 60-781 Poznan, Poland; mnowicki@ump.edu.pl; 2 Department of Neonatology, Biophysical Monitoring and Cardiopulmonary Therapies Research Unit, Poznan University of Medical Sciences, 33 Polna Street, 60-535 Poznan, Poland; tszczapa@gmail.com; 3 Department of Risk Group Dentistry, Chair of Pediatric Dentistry, Poznan University of Medical Sciences, 70 Bukowska Street, 60-812 Poznan, Poland; pn-mb-l@o2.pl (M.B.-L.); karolinagerreth@poczta.onet.pl (K.G.)

**Keywords:** early childhood caries, single nucleotide polymorphisms, artificial neural network, early prediction model, complex trait

## Abstract

Background: Several genes and single nucleotide polymorphisms (SNPs) have been associated with early childhood caries. However, they are highly age- and population-dependent and the majority of existing caries prediction models are based on environmental and behavioral factors only and are scarce in infants. Methods: We examined 6 novel and previously analyzed 22 SNPs in the cohort of 95 Polish children (48 caries, 47 caries-free) aged 2–3 years. All polymorphisms were genotyped from DNA extracted from oral epithelium samples. We used Fisher’s exact test, receiver operator characteristic (ROC) curve and uni-/multi-variable logistic regression to test the association of SNPs with the disease, followed by the neural network (NN) analysis. Results: The logistic regression (LogReg) model showed 90% sensitivity and 96% specificity, overall accuracy of 93% (*p* < 0.0001), and the area under the curve (AUC) was 0.970 (95% CI: 0.912–0.994; *p* < 0.0001). We found 90.9–98.4% and 73.6–87.2% prediction accuracy in the test and validation predictions, respectively. The strongest predictors were: *AMELX*_rs17878486 and *TUFT1*_rs2337360 (in both LogReg and NN), *MMP16*_rs1042937 (in NN) and *ENAM*_rs12640848 (in LogReg). Conclusions: Neural network prediction model might be a substantial tool for screening/early preventive treatment of patients at high risk of caries development in the early childhood. The knowledge of potential risk status could allow early targeted training in oral hygiene and modifications of eating habits.

## 1. Introduction

Dental caries is a chronic, multifactorial and dynamic disease that affects up to 83% of the global population, irrespective of the age. Early childhood caries (ECC) is defined as the presence of one or more decayed, missing or filled primary teeth in a child up to 71 months of age [1,2]. In Poland, based on the epidemiological studies, 53.8% of 3-year-old children have on average 2.4 effected teeth and this prevalence is higher when compared to the data from Western Europe [3,4]. However, even 6-month-old infants have been found to develop caries lesions; therefore, it has been suggested that an early caries prevention should start in the 4th month mother’s pregnancy [5]. Additionally, some mothers have presented reluctance to visit clinics regularly [6] and 48% of 3-year-old Polish children have never been to the dentist so far, although the dental care is free [4].

Interestingly, despite the treatment programs and caries prevention for preschoolers, about 15% of young patients would still develop the disease, which suggests an intrinsic, genetic factor influencing the individual host’s susceptibility to caries [7]. In addition, patients exposed to the same level of environmental factors and with comparable behavioral factors, might present distinct susceptibility to caries lesions [8]. Indeed, biological factors show stronger association with caries than the lifestyle and socio-economic factors, the latter only being responsible for intermediate and distant effects [9]. Genetic and immunological factors have been considered to be more important in enamel defects than the eating habits or nutritional deficits and the overall health status and immunodeficiencies in children have been shown to significantly affect the enamel hypomineralization [1]. For the last decade, several genes and genetic polymorphisms have been identified and associated with dental caries lesions in patients of different ages and ethnicities [1,7,10,11,12,13]. However, the majority of the existing caries prediction models lack biological, especially genetic factors. Selected and validated single nucleotide polymorphisms (SNPs) might be genotyped easily and early in a child’s life, and represent a potentially valuable tool for the caries risk prediction. This could allow enhanced early targeted prevention for infants and toddlers at greatest risk. As most of the eating and oral health habits can be changed at any time, this might result in better health and quality of life.

Our previous research [14,15] showed significant association of genetic polymorphisms with caries in 2–3-year-old Polish children. The aim of this study was to present caries prediction model based on chosen polymorphisms from all three studies, as predictors, using the artificial neural network approach.

## 2. Materials and Methods

### 2.1. Ethical Issues

The study was reviewed and approved by the Bioethical Committee of the Poznan University of Medical Sciences (resolutions no. 590/13, 605/14, and 727/18).

### 2.2. Study Design

In total, 262 children from the four nurseries in the central-west Poland were enrolled. In the two previous studies [14,15], we analyzed the differences in the frequencies of alleles and genotypes of 18 single nucleotide polymorphisms (SNPs) in 7 genes in the reference of caries experience in the cohort of Polish children. Another six SNPs in 6 genes with a presumable role in caries pathogenesis were analyzed in this study [1,10,16,17,18]. In brief, we genotyped: rs4547741 in *LTF*, rs7217186 in *ALOX15*, rs10429371 in *MMP16*, rs7096206 in *MBL2*, rs1884302 in *SMAD6* and rs1711437 in *MMP20*. Additional analyses (including additional statistics and machine learning techniques) were performed by combining SNP testing results with data from the previous studies to determine predictive capacity of studied variants for childhood caries. Two variants, i.e., rs2609428 and rs36064169 in *ENAM* had homozygous status in all individuals, therefore they were excluded from statistical analyses. The remaining 22 differentiated SNPs analyzed in this study are presented in Table 1.

### 2.3. Dental Examination

Dental examination was carried out by a trained and calibrated dentist (K.G.), specialist in pediatric dentistry, after calibration by an experienced specialist (M.B.-L.). The intra-examiner agreement was assessed by second dental examination in a group of 10 children after 2 weeks, with a κ of 1.00. Teeth evaluation was performed in the nursery school, with the use of a dental mirror and a probe, in an artificial light. The dentition was not additionally cleaned before examination. Assessment of the teeth concerned the occurrence of carious cavities as well as initial (incipient) caries lesions (non-cavitated lesions, white spot). All tooth surfaces accessible for examination were investigated. Dental examination concerned the occurrence of teeth with carious cavities (dt) and with initial (incipient) caries lesions (non-cavitated, white spot; di), i.e., the stage before cavitation during the process of dental caries development [19]. In accordance with the international standards, the white spot lesions were included in dental caries diagnosis [20,21] as they indicate the susceptibility of an individual to dental caries and are prevalent in primary dentition in children in the first years of life. White spot lesions were easily differentiated from developmental defects of enamel on the clinical ground based on the association between caries lesion and its location on the tooth and the areas of mature plaque [22]. However, when it was impossible or difficult to differentiate the white spot lesions from other changes in some individuals, they were excluded from examination and further analyses. Radiograph of the children’s dentition was not taken.

Detailed inclusion and exclusion criteria are presented in Table 2. Out of 262 individuals examined in the study, 48 children (18.3%) were diagnosed with caries and comprised a study group. Out of the rest 214 subjects we chose 48 sex- and age-matched individuals that comprised a control group. There were 23 males (48%) aged 20 to 42 months (mean 30.2 ± 6.2) and 25 females (52%) aged 20 to 40 months (mean 30.8 ± 5.7). One control sample had low DNA concentration and was fully utilized during two previous studies; therefore, it was not available for genotyping in this study and was excluded from statistical analyses. We intended to use the genotyping and distribution results, therefore the missing control sample was not replaced by another matching sample from the caries-free cohort in this study. Finally, the control group comprised 47 individuals, including 23 males (49%) aged 20 to 42 months (mean 31.2 ± 5.3) and 24 females (51%) aged 20 to 38 months (mean 28.3 ± 5.4).

### 2.4. Biological Samples and Genotyping

Biological material was obtained directly after dental examination. Samples were gathered using buccal swabs, which were provided for each child in sterile packs. The procedure included rubbing of the inside of the mouth, at least ten times, from each side of both cheeks in order to scrub epithelial cells with saliva. Subsequently, the swab was put inside the 1.5 mL Eppendorf tube, and the plastic stick was cut off. The tube was placed in a portable fridge at +4 °C until DNA extraction that was done the same day using EXTRACTME DNA Swab&Semen Kit (Blirt S.A., Gdansk, Poland) according to the manufacturer’s protocol and kept at −20 °C for further analyses. We used TaqMan probes (Applied Biosystems, ThermoFisher Scientific, Frederick, MD, USA) and 7900HT Fast Real-Time PCR System, according to the manufacturer’s instructions. Per each real-time PCR reaction, we used 10ng of genomic DNA that was previously extracted and stored at −20 °C. SDS v2.4 software was used to run the analysis and for allele calling.

### 2.5. Statistics

The continuous data were presented as mean ± standard deviation (SD) while the categorical data were counted and presented as numbers. The Kruskal–Wallis test was applied for comparison of the means of continuous data. We used chi square test for testing the Hardy–Weinberg Equilibrium and the Fisher’s exact test for estimating differences in allele and genotype frequencies between study subgroups and between study groups and CEU data (samples of Northern and Western European ancestry, from the International HapMap Project). The dominant (AA vs. Aa+aa), over-dominant (Aa vs. AA+aa), recessive (aa vs. AA+Aa) and allelic (A vs. a) models of genetic inheritance were applied [23]. Cochran–Armitage test for trend, the most common approach in case–control analyses, was used to test the additive model of inheritance [24]. The analyses were run using IBM SPSS Statistics software. Additionally, SHEsis software was applied for haplotype analysis.

### 2.6. Association and Prediction Analyses

For the modeling purposes, two approaches were assessed, namely logistic regression and artificial neural network. Firstly, we used univariable logistic regression to test the impact of individual variables, accompanied by the receiver operator characteristic (ROC) curve and area under the receiver operating characteristic (AUC) value to evaluate the sensitivity, specificity and the discriminatory ability of each factor. Statistical significance in univariable logistic regression, in ROC analysis, and/or in the Fisher’s exact test for single variables were applied as inclusion criteria for multivariable logistic regression and neural network analyses. Multivariable logistic regression was run using the enter method and the following characteristics were applied to describe the model: R^2^ Negelkerke and Cox and Snell R^2^ values to assess how well the model explains the data, the coefficient β and the exponentiated coefficient β (the odds ratio) values to indicate the relationship between each variable and the outcome, Hosmer and Lemeshow test to determine a goodness of fit of the data with the model, and, at last, the ROC curve to evaluate the predictive accuracy and the discrimination power of the model. IBM SPSS Statistics software v27.0.1.0 was used for logistic regression and ROC analyses and Statistica v13.3 were employed for neural network modeling.

Artificial neural network is a deep learning approach that, in the image of the human brain, self-learns from experience and adjusts to a situation. Briefly, neural network (NN) consists of multiple neurons, called nodes, and interconnections among them creating a complex structure, in which information passes imitating the system of real neurons. Each interconnection is given its weight, upon which the strength of the association is acquired. A typical network contains an input, hidden and output layers. The input layer receives the input signal to be processed, i.e., the data, while the hidden layer performs all the computational processes resulting in an outcome prediction in the output layer. The results are compared to the real observations and each time the process is repeated, until the smallest prediction error is reached. The great advantage of the NN approach is that it enables detecting complex nonlinear associations between variables and an outcome as well as all possible interactions between variables themselves, using multiple distinct learning algorithms, that are adjusted for the data type. We used the following parameters: a typical multilayer perceptron as a network type, 4 to 12 hidden layers (default), the Broyden–Fletcher–Goldfarb–Shanno (BFGS) algorithm as an iterative method, 4 types of activation functions, i.e., linear, logistic, exponential and tanh, and SOS (symbiotic organisms search) error rate type as a training method, that gives the best accuracy [25]. Out of the total data, 70%, 15% and 15% were used as training, testing and validation set, respectively. In total, 25 different models were created, out of which the 6 best, based on self-learning, were saved and analyzed in detail.

## 3. Results

### 3.1. Demographic Data

The caries rate in our cohort of preschool children was 18.3% (48/262) and the number of affected males was comparable with affected females (23 vs. 25). To minimize the potential demographic stratification, the control samples were adjusted to the caries samples in the reference to age and gender (*p* = 0.4163 and *p* = 0.9208, respectively). There was no difference between the number of erupted teeth or the type, i.e., incisors, canines, molars, between the groups (*p* = 0.3945, *p* = 0.3250, *p* = 0.7148, *p* = 0.1988, respectively). Additionally, the number of erupted teeth and its types did not differ between males and females, in caries or control group.

### 3.2. Genotyping

All markers genotyped in the present research were in Hardy–Weinberg equilibrium. The distribution of genotypes and alleles in six SNPs, in caries and control groups as well as in the study cohort and CEU data are presented in Table 3.

We spotted an almost 26-fold higher occurrence of rs10429371 recessive TT homozygote (*p* = 0.0262) and a 1.5-fold higher occurrence of the T allele (*p* = 0.1645) in caries patients in comparison with healthy controls, and the CT heterozygote was 2.5-fold more frequent (*p* = 0.0320) in the control group in comparison to caries group. In the case of another variant, rs7096206, the recessive GG homozygote and the G allele were 9.6-fold (*p* = 0.0363) and over 2-fold (*p* = 0.0180), respectively, more frequent in controls than in caries patients. The frequencies of alleles and genotypes for the rest of the genotyped SNPs did not differ between the groups or the results were statistically insignificant. When the subgroups were compared in reference to gender, none of the results were significant, although there was an almost 2-fold higher occurrence of rs10429371_T and TT variants in males, as well as over 2.5-fold higher occurrence of the wild CC homozygote in females, in caries patients, while no such observation was made for the controls. When our results were compared to the CEU data, we spotted significant differences for rs4547741, rs7217186 and rs10429371, and the latter SNP showed significance both for allele and genotype frequencies, showing the recessive T and TT variants to be, respectively, 8-fold and almost 18-fold more frequent in the CEU group than in our Polish cohort (*p* < 0.001 for both comparisons) (Table 3).

The differences in the frequency of the other 18 single nucleotide variants used for further statistical analyses in the present research were discussed in our previous studies, showing the association of recessive *ENAM* rs12640848_G, recessive *AMELX* rs17878486_T, wild *TUFT1* rs2337360_A and two recessive *KLK4* rs2235091_G and rs198969_G variants with caries outcome, and one recessive variant in *AMBN*, rs34538475_T, with the absence of the disease [14,15].

When the Cochran–Armitage test for trend was applied (see Table 4), out of eight significantly different distributed variants between the groups, six showed the positive significant additive model of inheritance and they were as follows: rs7096206, rs12640848, rs17878486, rs2337360, rs2235091 and rs198969. In turn, variants rs10429371 and rs34538475 showed stronger recessive modes of inheritance.

### 3.3. Haplotype Analysis

Although not each variant comprising the haplotype presented significance in single variant analysis, the overall haplotype analysis showed some association with the disease or disease-free trait. Briefly, *AMBN* TC and TT haplotypes (comprising rs34538475 and rs4694075, respectively) were significantly associated with healthy controls, while GC haplotype showed association with caries. Additionally, strong association with caries was observed for *TUFT1* AAA, AAG and AGG haplotypes (comprising rs3790506, rs4970957, rs2337360, respectively) and the significances for the rest of *TUFT1* haplotypes were most probably the result of quite low numbers of dominant and recessive homozygotes in both groups for rs3790506 and rs4970957, respectively, which was reflected by the absence of some haplotype variants in one group and their low frequency in the other. Likewise, significance was observed for rs2235091_rs198969 TC haplotype in *MMP20*, most probably due to its absence in of the study subgroups, and a low frequency in the other. Both SNPs in *KLK4* were significant in single locus analysis, however, only rs2235091_rs198969 GG haplotype showed significant association with caries, demonstrating that both recessive alleles were essential for caries effect and, analogically, the AC haplotype was significantly associated with non-disease trait, showing that both dominant alleles were essential for protective effect. Two haplotypes in *ENAM* were significantly associated with caries and non-caries trait, most possibly due to the middle allele of rs12640848, i.e., the only significant SNP in *ENAM* in other statistical test conducted. Haplotype CAAGA was over twofold more frequent in caries patients, corroborating the A allele as a risk variant, while haplotype CAGGA was almost 2.5-fold times more frequent in controls, which confirmed the G allele as a protective variant. The other four SNPs of *ENAM* did not contribute into the haplotype significance. No significant results were found for *TFIP11* haplotypes. The haplotype distributions are shown in details in Table 5.

### 3.4. Caries Association and Prediction

Table 6 includes the results of univariable logistic regression that depicted eight variables, that were all single nucleotide polymorphisms, i.e., rs10429371, rs7096206, rs12640848, rs17878486, rs12640848, rs2337360, rs2235091 and rs198969, to be significantly associated with caries outcome and those eight SNPs were then included in multivariable logistic regression analysis and neural network prediction. The overall multivariable logistic regression model characteristics are shown in Table 7 and information about single variables in the model are shown in Table 8.

**Figure 1:**
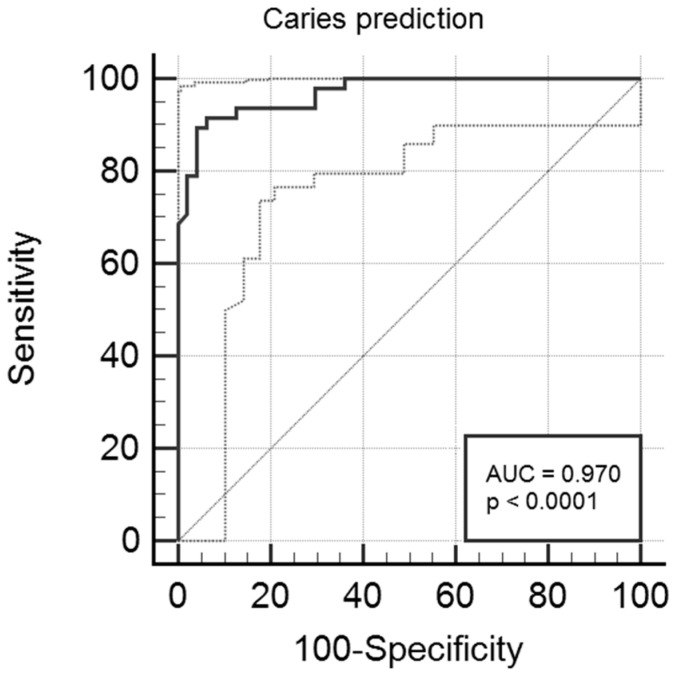
The receiver operator characteristic (ROC) curve analysis and area under the receiver operating characteristic (AUC) value. The solid black line indicates the ROC curve based on 8 variables, i.e., rs10429371, rs7096206, rs12640848, rs17878486, rs12640848, rs2337360, rs2235091 and rs198969, and the true positives (sensitivity) and false negatives (100-specificity) for caries prediction when compared to the actual data. The dashed lines indicate the ROC curves for 95% Confidence Interval.

Briefly, the strongest association with caries was assessed for rs17878486 in *AMELX*, rs2337360 in *TUFT1* and rs12640848 in *ENAM*, that remained in the final model at *p* < 0.05 (*p* = 0.0008, *p* = 0.0040, *p* = 0.0401, respectively). The model evaluation gave 93% total number of correct calls with 90% sensitivity and 96% specificity and a strong level of significance *p* < 0.0001. The AUC value of the model (0.970 (95% CI:0.912–0.994; standard error = 0.014), *p* < 0.0001) was also high. The ROC curve was constructed by plotting the true caries rate against the false caries rate and the prediction was made using all eight SNPs that were significantly associated with the outcome in single locus analyses. The rest of parameters, e.g., the prediction accuracy and goodness of fit, reached the high level of the overall performance of the model (see Table 7 and Figure 1).

According to other mathematical algorithms used in neural network analysis, all eight variables that performed at *p* < 0.05 in univariable analyses were also introduced in the NN model, although only three of them hold the significance in multivariable logistic regression. The overall performance of six neural network prediction models is presented in Table 9.

**Figure 2:**
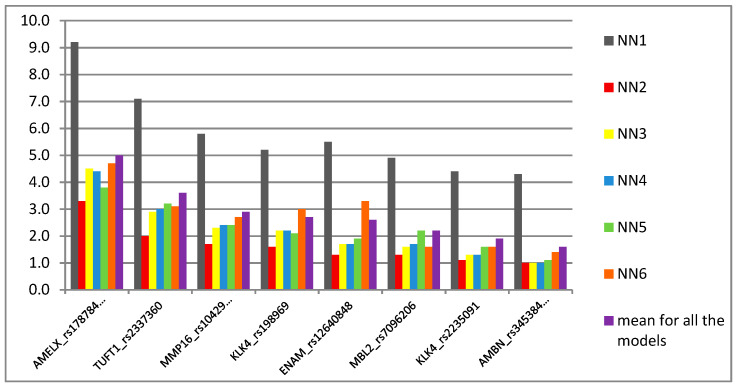
Prediction sensitivity for 8 SNPs used as predictors in neural network modeling. The higher the error rate value, the more sensitive the model is to the lack of a variable. The arrangement of the predictors in the figure is shown on the basis of the mean error value for the 6 neural network models (from NN1to NN6).

All the models reached a high prediction accuracy, from 90.9% to 98.4% in the test analysis. The best model, namely NN1, which gave the highest prediction accuracy in the test analysis (98.4%), simultaneously gave the lowest rate in the validation analysis (73.6%). The other five models performed relatively high in the validation analysis, i.e., 85.3–87.2%. All four types of activation function algorithms were used in the final prediction models. Only one model, NN3, used the linear function in the hidden layer, while logistic, exponential and tanh functions turned out to be better fitted, as they were used interchangeably in the rest of the models. In fact, the latter two functions support the backpropagation process, and therefore speed up the model’s self-training process, which is one of the crucial steps in a multilayer neural network system [26,27]. When analyzing the prediction sensitivity of single markers, described as their usefulness, interestingly, all of the eight markers turned out to be crucial for the prediction models. Figure 2 shows the overall importance of eight markers in six caries prediction models. The sensitivity of prediction allows distinguishing variables that are important from those that do not contribute, or contribute little, to the overall performance of a model, and therefore, can be rejected. The greater the error after rejection of a variable to the original error, the more sensitive the network model is to the lack of this variable. In this study, the top three predictors in the reference to each single model as well as to the mean value of error rate were: AMELX_rs17878486, TUFT1_rs2337360 and MMP16_rs1042937, while the least important predictor was AMBN_rs34538475. Nevertheless, all eight markers gave an error value above “0”, which means that even the weaker one was still important for the caries prediction.

## 4. Discussion

We presented a complex analysis of 22 differentiated single nucleotide polymorphisms in prediction of dental caries in primary dentition of children. This study is an extended analysis utilizing additional data from our previous studies [14,15]. Deep learning neural network models for caries prediction were applied in a homogeneous cohort of 2–3-year-old children living in an urban environment under similar cultural conditions.

The previous caries experience, independent of an individual’s age, has previously been reported as the strongest and the most universal risk factor for future caries development [28]. However, it is challenging to assess the previous caries experience in infants and toddlers. Most of the previous studies and distinct caries prediction models apply to adults, school children or older preschoolers, while models for toddlers are scarce [29]. Another obstacle in assessing well-performing caries prediction model are discrepancies among the studies, i.e., imprecise definition of caries phenotype and caries lesions or inconsistency in the terms used by the researchers [9,23,30,31]. Furthermore, the majority of acquired caries prediction models are based on demographical and environmental factors and the only biological feature that has been considered is the cariogenic bacteria colonization of oral cavity.

The first linkage studies in caries were performed in 2008 [32] and the first genome-wide association studies for early childhood caries were published in 2011 [33]. Abbasoğlu et al. [10] were the first to correlate environmental and genetic factors in ECC in Turkish 2–5-year-olds, while Lewis et al. [34] considered several single nucleotide polymorphisms, however they were not included in the final caries prediction meta-analysis. Another technical obstacle in any caries prediction model implementation in practice is an almost total lack of replication and validation studies in independent populations [13,35]. Mejare et al. [28] depicted 17 studies on caries prediction in preschoolers, defined as children of age <1 to 6 years old. None of them used genetic variables as prediction factors, most gave moderate prediction accuracy results and only one study by Holgerson et al. [36] based on environmental factors and saliva sampling in 2-year-olds was validated. Persistent high dental caries rate in the general Polish population and nearly constant prevalence and severity index of the disease in children in the recent years [37] should encourage both researchers and clinicians to develop better and validated prediction approaches that might be implemented in practice.

In this study, we obtained relatively high prediction accuracy ranging from 90.9% to 98.4%, depending on the prediction model, using a neural network approach. Additionally, multivariate logistic regression analysis showed accuracy of 93% with high sensitivity and specificity values, i.e., 89.6% and 95.7%, respectively. Results of both approaches were gender- and age-independent, as the two study subgroups were adjusted for both features. The most important predictors/indicators in both assays were *AMELX* rs17878486 and *TUFT1* rs2337360 [15]. In brief, *AMELX* and *TUFT1* are the genes that play a crucial role in the enamel formation process and the single nucleotide polymorphisms have previously been attributed to high caries susceptibility, in primary as well as permanent dentition, although the risk allele varied depending on population and age [12,30,38]. Another factor that was significantly associated with caries outcome in both uni- and multivariable logistic regression was *ENAM* rs12640848. *ENAM* encodes enamel-special protein enamelin that is a critical factor during enamel maturation. Our results were in agreement with other reports, supporting the presumable role of rs12640848_G as a protective factor in ECC [10,30]. Interestingly, when the SNP was used in this study as a predictor in neural network analysis, it turned out to be an intermediate marker in the scale of importance (Figure 2). This might be explained by the fact that both wild AA and rare GG genotypes were associated with the opposite outcomes, i.e., caries and caries-free phenotype, respectively. Conversely, another SNP, i.e., *MMP16* rs10429371, was the third most important predictor in NN models, although it did not hold the significance as an indicator in multivariate analysis. This is an interesting finding, since rs10429371 explained over 20% of the trait in single locus analysis and the TT variant was nearly 26 times more frequent in caries individuals when compared to controls, showing a strong recessive pattern of inheritance and suggesting TT as a risk variant for dental caries development in the studied individuals. Additionally, it was one of the variants for which alleles and genotypes were differently distributed in the cohort in this study in comparison to the CEU individuals in favor of wild C and CC variants (Table 3). Matrix metalloproteinases (MMPs) play an important role in early tooth development by regulating ameloblast maturation and formation of enamel. Several types of MMPs have been described to be involved in dentin collagen degradation and dental caries lesion progression [12,34,39,40,41,42]. *MMP16* rs10429371 was previously associated with caries in white adults [34]. Linhartova et al. [41] observed higher incidence of rare T allele in caries children aged 13–15 years, although it did not reach the statistical significance level.

The second variant that showed significant association with caries outcome and was genotyped in this study was rs7096206 in *MBL2*. Mannose binding protein (MBL2) is an acute phase protein that plays an important role in innate immunity. The study showed that *MBL2* polymorphisms are involved in several infectious diseases and the top ranked rs7096206 has been annotated as deleterious to the protein’s function and described as one of the most functionally important variants in the gene [42]. Rs7096206_G was found to be a risk factor in Polish 5-year-olds in reference to higher vs. lower caries experience, while it had no effect in 12-year-olds [1]. Interestingly, the same rare variant was significantly associated with no caries experience in 2–3-year-olds in our study, showing an additive model of inheritance. On the other hand, in the former study [1], rs7096206_G was associated with higher caries experience in both age subgroups while in the haplotype with rs1800450_G, and the CG haplotype had the opposite effect. Alike, rs7096206 genotype distribution was insignificant, but in haplotype with rs7501477_T it correlated with caries experience in Saudi 5–13-year-old children [43]. It might suggest that other SNPs could be associated with a more complex pattern of the disease and/or that distinct genetic variants could be involved at different ages. Such differences, i.e., in primary vs. permanent dentition, were also acknowledged by Wang et al. [44]. Likewise, as the rs7096206_G variant is associated with lower MBL2 serum levels predisposing to infections [45], other SNPs could show strong linkage disequilibrium accounting for possible protective mechanisms in the toddlers in our study. Nevertheless, the SNP was not significant in multivariate analysis, despite the drastic OR value, i.e., 3.36 × 10^−9^.

We did not observe any association of other SNPs in MMP, i.e., *MMP20* rs1711437 genotyped in this study and rs1784418 in the previous study [15], with caries or caries-free phenotype. MMP20 (enamelysin) is the early protease secreted during enamel development and is involved in both dentin and enamel decomposition [46]. Only Antunes et al. [47] found both variants to be associated with early childhood caries. Yet, the results of other studies of *MMP20* SNPs were on the border of significance in 5-year-old Caucasian children with dental caries or have been associated more with poor oral hygiene and dietary habits than the disease itself in 5–14-year-old Caucasians [16,46]. Likewise, rs45447741 in *LTF*, rs7217186 in *ALOX15* and rs1884302 in *SMAD6* presented no association with caries experience in this study in any of the statistical tests used.

The rest of significantly different distributed variants in the previous study [15], i.e., *AMBN* rs34538475 and *KLK4* rs2235091and rs198969, were not significant in multivariate analysis and their importance in caries prediction models were of a moderate (rs2235091and rs198969) to low (rs34538475) degree (Figure 2). *KLK4* plays an important role in the late stages of enamel development, while *AMBN*, together with *AMELX*, is crucial for enamel matrix formation and mineralization. The roles of the abovementioned SNPs in both genes as risk factors in caries development in children were partially in agreement with other authors, depending on studied population and children’s age [10,12,46]. Additionally, the haplotype analysis showed that alleles of both variants in *KLK4* were necessary for the risk (i.e., rs2235091G_rs198969G haplotype) and protective (i.e., rs2235091A_rs198969C haplotype) effect.

It must be emphasized that the differences in association of genetic variants with caries experience and severity in the studies occurs not only due to the differences between the populations but also in one population itself, even from one individual to another [34]. Single nucleotide polymorphisms are often highly variable between distinct ethnic cohorts and, at least partially, resemble divergence in human phenotypes, including different disease susceptibility or drug response. Dental caries is a highly complex trait, also in reference to environmental factors, i.e., socioeconomic and cultural factors, age, oral hygiene, eating habits, also the course of pregnancy and mother–child relations [3,6,46,48,49,50]. Age appears one of the key factors in caries analysis, as the disease experience is differentially defined in reference to the age of patients. Some authors emphasize that each age group should be characterized by other sets of temporal variables [50], with the most important risk factors as follows: in 2-year-olds—allergies and infections before first tooth eruption and intake of drugs during the first 12 months of life, in 3-year-olds—mother’s age at the time of pregnancy and smoking during pregnancy [29], consumption of sweetened food during first 12 months of life and nocturnal drinking of sweet drinks above 12th month [3], in 4-year-olds and older children—frequency of tooth brushing, fluoride treatment and mother’s education [51].

One of the hypotheses might be that distinct processes, and hence different genes and polymorphisms, play a significant role throughout the stages of a child’s growth and development. Since the genetic sequence does not change over the course of life, each stage might be sensitive to distinct variants, including those that shape susceptibility to caries development. Simultaneously, the sensitivity to the secondary environmental factors and the tertiary behavioral factors, responsible for the intermediate and distal effects, respectively, might also alter. Substantially, it can be explained by the order of teeth eruption, their type and number. Caries lesions appear first on incisors and molars, also maxillary teeth are more often affected than mandibular teeth. Caries formation predominantly affects teeth with deep and/or narrow pits and fissures on the occlusal surface, which is closely related to the morphology of primary dentition and molars [4,46,50]. The deeper and narrower the pit, the easier it is for the bacterial plaque to penetrate and adhere to a tooth surface. The higher the number of teeth with deep and narrow pits, together with inappropriate brushing, the more frequent the predisposition to higher caries rate, which is especially relevant in preschoolers [50,52,53]. The similar pattern of caries development has been observed in 3-year-old children in other studies [53,54,55]. In this study, we did not observe differences in the total number of erupted teeth and the number of the teeth types was similar between affected and caries-free children and between boys and girls in both subgroups (not shown). Although we did not conduct a follow-up study, some authors pointed that caries susceptibility varies depending on the tooth morphology and rises sharply after 2–3 years after the tooth eruption, when posteruptive enamel maturation takes place [4,5]. Therefore, dental caries seems to be a remarkably divergent trait constantly changing in time and with its occurrence increasing with age. The prevalence of caries experience in our study was 18.3% and was comparable to children of the corresponding age of other ethnicities, i.e., Western Europe, Eastern-Southern Asia and Sub-Saharan Africa [3,29,56]. Interestingly, it appeared to be much lower when compared to other studies concerning Polish children with active caries of distinct regions, i.e., 40.8% in the Podlasie region [57], 53.8% in Lower Silesian, Malopolskie and Lubelskie voivodships [4] or from 35% to 56.6% in the general 2–3-year-olds population [3,13,57]. However, according to Werneck et al. [13] caries rate can reach even 85% in preschoolers.

Another risk factor for caries lesions development, closely related to children’s age, is the presence and composition of bacterial plaque in the host. The level of colonization by cariogenic bacteria has been considered the strongest risk factor in 3-year-old Polish children [49], however, it should be emphasized that it might be influenced by other features. Firstly, the activity of Lactobacilli and Mutans Streptococci is higher in preschoolers and in primary dentition in comparison with older children and permanent teeth [10,50,58], therefore it might be a biological predisposition of the host. Secondly, the younger the child, the more attention and supervision over teeth brushing should be provided by the parents/guardians, therefore minimizing the bacterial film and setting a good hygiene habits. The mother not brushing a child’s teeth was found a major risk factor in Mutans Streptococci infections and ECC in Australian children aged 12–72 months but also in 9-month-old Thai children [6,59].

Both genetic and host factors fluctuate with age, and with a broad spectrum of environmental factors, they contribute to the development of caries lesion differently in every individual. Moreover, even children of the same ethnicity that are exposed to the same levels of risk factors might present distinct caries severity index and patterns of the disease [13], which strongly supports the existence of other, intrinsic, i.e., genetic components. Still, depending on the population and its habits, prediction models using the same risk factors might be characterized by distinct final prediction parameters, as well as comparable prediction features of different models might be determined by distinct input variables [8]. To mention a few, Kalhan et al. reached a high AUC value of 0.81–0.91 of caries prediction in 2-year-olds and 0.79 in 3-year-olds [29], while Fontana et al. [60] assessed AUC of 0.73 in 4-year-old children. Tamaki et al. [61] came across 33 studies on caries prediction, however, the majority of them used logistic regression analyses and only some authors used additional, advanced machine learning algorithms to compare the results, namely artificial neural network, decision analysis or classification and regression trees [27,50,61,62,63]. The benefit of logistic regression analysis is that it can be conducted using non-complicated software; however, the procedure is descriptive in its nature and should be applied to assess risk indicators, not predictors. On the other hand, advanced machine learning methods consider the unequal strength of potential markers, so that a weak factor is not hidden by the strong one and predictive power is more reliable [9,51]. In fact, we observed some differences in the importance level of studied variants between the two approaches, although it is not entirely adequate to compare the results of different algorithms, even describing the same features. Still, one of NN models—NN5, in which logistic function was applied as an activation algorithm in both hidden and output layers, gave the most outstanding importance values of all tested predictors, while in the remaining 5 models based on distinct algorithms were lower but comparable with each other within one model. The NN5 model presented the second-best prediction accuracy in this study; however, using logistic methods only might suggest over-fitting of the model, even in advanced machine learning approaches. Likewise, So and Sham [64] stated that ROC curve analysis is not directly correlated with the disease risk and even high significance, i.e., low *p*-values, is not equal to a good predictive power. Javed et al. [27] obtained 99% accuracy in caries prediction in 6–14-year-old Indian children using neural network modeling, and it was the method of choice in other studies with medical prediction [65,66,67,68].

Another issue worth mentioning is the nature of variables themselves. When studying some environmental, behavioral and biological factors, a collinearity might occur, e.g., the presence of dental plaque might be the result of a poor oral hygiene, which in turn might be correlated with a lack of guardians’ control over child’s brushing. [63]. While it is not a problem in advanced modeling approaches, it should be avoided in logistic regression analyses, since it tends to cause over-fitting of the data and spurious results [63,69]. The strength of this study is a homogeneous study group, that is sex- and age-adjusted, since both factors are found to be potential confounders in case–control studies [24]. Secondly, the assessment and comparison of the results and performance of two different statistical approaches, adjusted for the data and therefore more reliable, appears to be crucial when implementing the method into the clinics. The obvious limitation of this study and a necessary future direction is a larger study group and validation of the model.

The polymorphisms analyzed in this study and a high caries prediction rates indicate a strong genetic component in the course of the disease. Nevertheless, one has to remember about the multifactorial nature of caries and that even the best prediction model cannot fully describe the real life scenarios and neither solely genetic nor solely environmental factors can completely explain the disease cause. Hence, analyses exploring environmental and genetic predictors need to be conducted very carefully. Firstly, some genetic variants might influence behavioral habits, e.g., SNPs in taste genes [11] as well as non-genetic factors themselves might be correlated with one another, yielding spurious results. Secondly, compared to the environmental impact, if some genetic effect on a disease trait exists, it remains the main and unchangeable element in the disease incidence, especially early in life [70,71]. Even being under the influence of the environment, genetic factors combined with non-genetic findings have been replicated only partially and with the lack of statistical power [70]. Although many rare variants or the ones with smaller effect might be missed in purely genetic case–control studies, the subgroup analyses and high homogeneity of the study subgroups greatly improves the prediction [72,73]. Some authors [70,74] have studied and described interesting gene–environment association tests, that successively and carefully analyze both components in multiple testing approaches. McAllister et al. [75] have developed novel statistical approaches to detect genetic–non-genetic interactions with regard to different durations of exposure to environmental factors. Therefore, a fully developed caries prediction model undoubtedly requires a complex analysis of multiple factors as well as replication studies before implementation in practice. Still, the high prediction value of genetic polymorphisms presented in this study comprises valuable findings on early caries development that sets the direction of the future research.

## 5. Conclusions

In conclusion, our study demonstrates neural network models of high accuracy for dental caries prediction in early childhood, based solely on the genetic single nucleotide polymorphisms selected using more basic and less statistically advanced methods. An effective and early prediction of intrinsic risk factors might influence the change in eating habits, improve oral hygiene and other behavioral factors when an individual is at a high risk of developing caries lesions. Early implementation of preventive strategies and a customized early treatment might decrease the risk of caries while improving children’s health, the quality of life and self-esteem, as well as reducing the financial burden associated with medical care.

## Figures and Tables

**Table 1:** Twenty-two differentiated SNPs analyzed in the current study.

SNPs Analyzed in This Study					
SNP ID	Gene	Gene ID ^1^	Chromosome: Location	Type of Variant	AMINO Acid Change
rs4547741	*LTF (Lactotransferrin)*	4057	3:46458968	intron variant	C>T
rs7217186	*ALOX15 (Arachidonate 15-Lipoxygenase)*	246	17:4636097	intron variant	C>T
rs10429371	*MMP16 (Matrix Metallopeptidase 16)*	4325	8:88981259	intron variant	C>T
rs7096206	*MBL2 (Mannose Binding Lectin 2)*	4153	10:52771925	intron variant	C>G
rs1884302	*SMAD 6 (SMAD Family Member 6)*	650	20:7125642	intron variant	C>T
rs1711437	*MMP20 (Matrix Metallopeptidase 20)*	9313	11:102594495	intron variant	C>T
rs1784418	*MMP20 (Matrix Metallopeptidase 20)*	9313	11:102613665	intron variant	C>T
rs17878486	*AMELX (Amelogenin X-Linked)*	265	X:11295828	intron variant	C>T
rs34538475	*AMBN (Ameloblastin)*	258	4:70605459	intron variant	G>T
rs4694075	*AMBN (Ameloblastin)*	258	4:70601197	intron variant	C>T
rs3790506	*TUFT1 (Tuftelin 1)*	7286	1:151565890	intron variant	A>G
rs4970957	*TUFT1 (Tuftelin 1)*	7286	1:151544912	intron variant	A>G
rs2337360	*TUFT1 (Tuftelin 1)*	7286	1:151542127	intron variant	A>G
rs134136	*TFIP11 (Tuftelin Interacting Protein 11)*	24144	22:26503508	intron variant	C>T
rs5997096	*TFIP11 (Tuftelin Interacting Protein 11)*	24144	22:26499991	intron variant	C>T
rs2235091	*KLK4 (Kallikrein Related Peptidase 4)*	9622	19:50907215	intron variant	A>G
rs198969	*KLK4 (Kallikrein Related Peptidase 4)*	9622	19:50910072	intron variant	C>G
rs7671281	*ENAM (Enamelin)*	10117	4:70643369	missense variant C>T, ^2^ NM_031889.3:c.1943T>C, ^3^ NP_114095.2:p.Ile648Thr	C>T
rs3796704	*ENAM (Enamelin)*	10117	4:70643714	missense variant G>A, NM_031889.3:c.2288G>A, NP_114095.2:p.Arg763Gln	A>G
rs12640848	*ENAM (Enamelin)*	10117	4:70640695	intron variant	A>G
rs144929717	*ENAM (Enamelin)*	10117	4:70632586	intron variant	G>A
rs139228330	*ENAM (Enamelin)*	10117	4:70635957	intron variant	A>G

Abbreviations: SNP—single nucleotide polymorphism; ID—identification; A—adenine; C—cytosine; G—guanine; T—thymine; Ile—isoleucine; Thr—threonine; Arg—arginine; Gln—glutamine; ^1^ Gene ID is an accession number from ncbi database (https://www.ncbi.nlm.nih.gov/, accessed on 17 January 2021); ^2^ NM_—prefix for nucleotide position in the reference sequence accessions for mRNA, c.—coding; ^3^ NP_—prefix for amino acid position in the reference sequence accessions for protein, p—protein.

**Table 2:** Inclusion and exclusion criteria.

Inclusion Criteria	Exclusion Criteria
**Study and control group**	
Age between 20 and 40 months	Age under 20 and over 40 months old
Group with the same ethnic, regional, cultural, or demographic origin	Other ethnic, regional, cultural, or demographic origin
Parental written and informed consent for dental check-up and oral swab collection	No parental written and informed consent for dental check-up and oral swab collection
Child’s cooperativeness	Child’s uncooperativeness
Set of properly filled in child’s dental chart and sample for molecular analysis	Lack of set of properly filled in child’s dental chart and sample for molecular analysis
Individuals from four nursery schools situated in the city of Poznan (Wielkopolska Province, central-west Poland) that constitute one institution	Individuals from other nursery schools than those selected to the research
Children present at nursery school on days of examination	Children absent at nursery school on days of examination
From 11 to 20 erupted primary teeth present in the oral cavity	Less than 11 primary teeth present in the oral cavity
Caucasian origin	Other than Caucasian origin
**Study group**	
Dental caries present in child’s dentition	Lack of dental caries in child’s dentition
**Control group**	
Lack of dental caries in child’s dentition	Dental caries present in child’s dentition

**Table 3:** Genotype and alleles distribution differences for 6 SNPs genotyped in this study between study subgroups (caries vs. caries-free) and between our cohort and CUE data.

Gene	SNP ID	Genotypes	Caries	Control	Test/Model ^1^	OR [95% CI]	*p*-Value	CEU data	
		Alleles	n (Freq)	n (Freq)				OR [95% CI]	*p*-Value
*LTF*	rs4547741	CC	35 (0.73)	32 (0.68)	CC vs. CT+TT	1.3 [0.5–3.1]	0.6059	0.5 [0.2–0.9]	0.0288 *
		CT	12 (0.25)	14 (0.3)	CT vs. CC+TT	0.8 [0.3–1.9]	0.6012	2 [0.9–3.9]	0.0605
		TT	1 (0.02)	1 (0.02)	TT vs. CC+CT	1.0 [0.1–16.1]	0.9880	5.3 [0.3–112.3]	0.2826
		
		C	82 (0.85)	78 (0.83)	T vs. C	0.8 [0.4–1.8]	0.6452	2.1 [1.1–4.1]	0.0209 *
		T	14 (0.15)	16 (0.17)					
*ALOX15*	rs7217186	CC	13 (0.27)	13 (0.28)	CC vs. CT+TT	1.0 [0.4–2.4]	0.9498	2.1 [1–4.3]	0.0395 *
		CT	23 (0.48)	21 (0.44)	CT vs. CC+TT	1.1 [0.5–2.6]	0.7519	0.7 [0.4–1.3]	0.2523
		TT	12 (0.25)	13 (0.28)	TT vs. CC+CT	0.9 [0.4–2.2]	0.7686	0.8 [0.4–1.5]	0.5382
		
		C	49 (0.51)	47 (0.5)	T vs. C	1.0 [0.5–1.7]	0.8858	0.7 [0.5–1.1]	0.1101
		T	47 (0.49)	47 (0.5)					
*MMP16*	rs10429371	CC	18 (0.37)	17 (0.36)	CC vs. CT+TT	1.1 [0.5–2.4]	0.8931	18.7 [5.5–63.4]	<0.001 ***
		CT	20 (0.42)	30 (0.64)	CT vs. CC+TT	0.4 [0.2–0.9]	0.0320 *	2.7 [1.5–4.8]	0.0011 **
		TT	10 (0.21)	0 (0.00)	TT vs. CC+CT	25.9 [1.5–456.4]	0.0262 *	0.1 [0.03–0.1]	<0.001 ***
		
		C	56 (0.58)	64 (0.68)	T vs. C	1.5 [0.8–2.8]	0.1645	0.1 [0.1–0.2]	<0.001 ***
		T	40 (0.42)	30 (0.32)					
*MBL2*	rs7096206	CC	31 (0.65)	23 (0.49)	CC vs. CG+GG	1.9 [0.8–4.3]	0.1254	0.9 [0.5–1.7]	0.8095
		CG	16 (0.33)	16 (0.34)	CG vs. CC+GG	1.0 [0.4–2.3]	0.9417	0.8 [0.5–1.5]	0.4959
		GG	1 (0.02)	8 (0.17)	GG vs. CC+CG	0.1 [0.01–0.9]	0.0363 *	3.5 [0.9–12.8]	0.0768
		
		C	78 (0.81)	62 (0.66)	G vs. C		0.0180 *	1.3 [0.8–2]	0.3473
		G	18 (0.19)	32 (0.34)					
*SMAD6*	rs1884302	CC	9 (0.19)	6 (0.13)	CC vs. CT+TT	1.6 [0.5–4.8]	0.4263	2.5 [1–6.4]	0.0616
		CT	21 (0.44)	21 (0.45)	CT vs. CC+TT	1.0 [0.4–2.2]	0.9272	0.7 0.4–1.3]	0.3091
		TT	18 (0.37)	20 (0.42)	TT vs. CC+CT	0.8 [0.4–1.8]	0.6154	0.9 [0.5–1.7]	0.8412
		
		C	39 (0.41)	33 (0.35)	T vs. C	0.8 [0.4–1.4]	0.4334	0.8 [0.5–1.2]	0.2969
		T	57 (0.59)	61 (0.65)					
*MMP20*	rs1711437	CC	19 (0.4)	20 (0.43)	CC vs. CT+TT	0.9 [0.4–2.0]	0.7686	1.3 [0.7–2.3]	0.4143
		CT	23 (0.48)	23 (0.49)	CT vs. CC+TT	1.0 [0.4–2.1]	0.9208	1 [0.6–1.8]	0.9929
		TT	6 (0.12)	4 (0.08)	TT vs. CC+CT	1.5 [0.4–5.8]	0.5287	0.6 [0.3–1.4]	0.2526
		
		C	61 (0.64)	63 (0.67)	T vs. C	1.2 [0.6–2.1]	0.6146	0.8 [0.5–1.2]	0.2497
		T	35 (0.36)	31 (0.33)					

The total number of individuals with caries *n* = 48, and controls *n* = 47. Abbreviations: SNP—single nucleotide polymorphism; ID—identification; OR—odds ratio; 95% CI—the 95% Confidence Interval; CEU—Utah residents with Northern and Western European ancestry, from the International HapMap Project; LTF—Lactotransferrin; ALOX15—Arachidonate 15-Lipoxygenase; MMP16—Matrix Metallopeptidase 16; MBL2—Mannose Binding Lectin 2; SMAD6—SMAD Family Member 6; MMP20—Matrix Metallopeptidase 20; C—cytosine; G—guanine; T—thymine.^1^ The odds ratio (OR) values with corresponding *p*-values were assessed by the Fisher’s exact test for the inheritance models: dominant AA vs. AB+BB; over-dominant AB vs. AA+BB; recessive BB vs. AA+AB; allelic A vs. B. *p*-values: *p* < 0.05 *, *p* < 0.01 **, *p* < 0.001 ***; all significant results are shown in bold.

**Table 4:** Cochran–Armitage test for trend analysis for 22 differentiated single nucleotide polymorphisms analyzed in this study.

SNP ID	Cochran–Armitage Test for Trend	
	Chi^2^	*p*-Value
rs4547741	0.219	0.6400
rs7217186	0.019	0.8898
rs10429371	2.234	0.1350
**rs7096206**	5.063	0.0244 *
rs1884302	0.579	0.4469
rs1711437	0.272	0.6019
**rs17878486**	35.991	<0.0001 ***
rs34538475	1.779	0.1823
rs4694075	2.494	0.1143
rs3790506	1.510	0.2191
rs4970957	1.885	0.1698
**rs2337360**	23.599	<0.0001 ***
rs134136	0.230	0.6316
rs5997096	0.130	0.7182
**rs2235091**	6.782	0.0092 **
**rs198969**	9.586	0.0020 **
rs1784418	0.584	0.4447
rs7671281	0.0013	0.9712
rs3796704	0.0492	0.8245
**rs12640848**	11.343	0.0008 ***
rs144929717	0.0492	0.8245
rs139228330	0.0492	0.8245

Abbreviations: SNP—single nucleotide polymorphism; ID—identification; significant *p*-value indicates additive model of inheritance, i.e., the possibility of heterozygote advantage. *p*-values: *p* < 0.05 *, *p* < 0.01 **, *p* < 0.001 ***; all significant results are shown in bold.

**Table 5:** Haplotypes distribution in caries and caries-free individuals in this study.

SNPs (Gene)	Haplotypes	Frequency		OR [95% CI]	*p*-Value
		Caries	Controls		
rs34538475_rs4694075	GC	0.503	0.108	8.4 [3.9–18.0]	<0.0001 *** (3.86 × 10^−9^)
(*AMBN*)	GT	0.236	0.200	1.2 [0.6–2.5]	0.5501
	TC	0.038	0.317	0.1 [0.03–0.3]	<0.0001 *** (4.65 × 10^−7^)
	TT	0.222	0.374	0.5 [0.3–0.9]	0.0220 *
rs2337360_rs4970957_rs3790506	A A A	0.129	0.294	0.4 [0.2–0.8]	0.0054 **
*(TUFT1)*	A A G	0.412	0.265	2.0 [1.1–3.6]	0.0314 *
	A G G	0.284	0.037	10.21 [3.2–32.4]	<0.0001 *** (4.01 × 10^−6^)
	G A G	0.074	0.197	0.3 [3.2–32.4]	0.0131 *
	G G A	0.000	0.015	-	0.2322
	G G G	0.000	0.192	-	<0.0001 *** (6.36 × 10^−6^)
	A G A	0.039	0.000	-	0.0547
	G A A	0.061	0.000	-	0.0147 *
rs134136_rs5997096	CC	0.573	0.606	0.9 [0.5–1.0]	0.6392
*(TFIP11)*	TC	0.302	0.287	1.1 [0.6–2.0]	0.8225
	TT	0.125	0.106	1.2 [0.5–2.9]	0.6884
rs2235091_rs198969	AC	0.432	0.670	0.4 [0.2–0.7]	0.0007 ***
*(KLK4)*	AG	0.181	0.118	1.7 [0.7–3.8]	0.2191
	GC	0.108	0.075	1.5 [0.6–4.1]	0.4273
	GG	0.288	0.138	2.5 [1.2–5.8]	0.0118 *
rs7671281_rs3796704_rs12640848_rs144929717_rs139228330	CAAGA	0.500	0.309	2.2 [1.2–4.1]	0.0072 **
*(ENAM)*	CAGGA	0.437	0.649	0.4 [0.2–0.8]	0.0035 **
	TGAAG	0.041	0.043	1.0 [0.2–4.0]	0.9644
	TGGAG	0.000	0.000	-	-
	CAAAG	0.011	0.000	-	0.3167
	TGAGA	0.000	0.000	-	-
	TGGGA	0.010	0.000	-	0.3234
rs1711437_rs1784418	CC	0.614	0.617	1.0 [0.6–1.8]	0.9614
*(MMP20)*	CT	0.022	0.053	0.4 [0.1–2.0]	0.2540
	TT	0.322	0.330	1.0 [0.5–1.8]	0.9081
	TC	0.043	0.000	-	0.0432 *

The total number of individuals with caries n=48, and controls n=47. Abbreviations: SNPs—single nucleotide polymorphisms; OR—odds ratio; 95% CI—the 95% Confidence Interval; AMBN—Ameloblastin; TUFT1—Tuftelin 1; TFIP11—Tuftelin Interacting Protein 11; KLK4—Kallikrein Related Peptidase 4; ENAM—enamelin; MMP20—Matrix Metallopeptidase 20; A—adenine, C—cytosine; G—guanine; T—thymine; *p*-values: *p* < 0.05 *, *p* < 0.01 **, *p* < 0.001 ***; all significant results are shown in bold.

**Table 6:** The performance of univariable logistic regression analysis.

Predictor	Coefficient of Determination R^2^	*p*-Value	AUC [95% CI]	*p*-Value	Sensitivity/Specificity/Total Number of Correct Calls	Statistical Significant Genotypes Distribution ^1^
rs4547741	0.0039	0.8703	0.525 [0.420–0.628]	0.617	72.9%/31.9%/52.6%	-
rs7217186	0.0201	0.6964	0.524 [0.419–0.627]	0.891	48%/55.3%/51.6%	-
rs10429371	0.2054	0.0004 ***	0.560 [0.544–0.744]	0.268	58.3%/63.8%/61.1%	+
rs7096206	0.0852	0.0435 *	0.599 [0.494–0.699]	0.0531	64.6%/51.1%/57.9%	+
rs1884302	0.0098	0.7051	0.539 [0.434–0.642]	0.4742	62.5%/42.6%/52.6%	-
rs1711437	0.0059	0.8115	0.525 [0.420–0.628]	0.6426	12.5%/91.5%/51.6%	-
rs17878486	0.4695	<0.0001 ***	0.830 [0.739–0.899]	<0.0001 ***	62.5%/93.6%/77.9%	+
rs34538475	0.0327	0.1298	0.571 [0.465–0.672]	0.1253	35.4%/78.7%/56.8%	+
rs4694075	0.0299	0.1423	0.582 [0.476–0.682]	0.1404	75%/38.3%/56.8%	-
rs3790506	0.0212	0.2177	0.559 [0.453–0.660]	0.2661	58.3%/51.1%/54.7%	-
rs4970957	0.0264	0.1683	0.580 [0.475–0.681]	0.1237	62.5%/55.3%/59%	-
rs2337360	0.3813	<0.0001 ***	0.781 [0.685–0.860]	<0.0001 ***	75%/89%/77.9%	+
rs134136	0.0032	0.6314	0.527 [0.422–0.630]	0.6251	68.8%/36.2%/52.6%	-
rs5997096	0.0026	0.6669	0.519 [0.414–0.622]	0.6699	25%/78.7%/51.6%	-
rs2235091	0.1317	0.0072 **	0.625 [0.520–0.723]	0.0152 *	20.8%/97.9%/59%	+
rs198969	0.1635	0.0020 **	0.658 [0.554–0.752]	0.0017 **	70.8%/51.1%/61.1%	+
rs1784418	0.0050	0.5498	0.535 0.430–0.638]	0.5168	41.7%/66%/53.7%	-
rs7671281	0.0014	0.7509	0.510 [0.405–0.614]	0.7533	100%/0%/50.5%	-
rs3796704	0.0014	0.7509	0.510 [0.405–0.614]	0.7533	100%/0%/50.5%	-
rs12640848	0.2016	0.0004 ***	0.672 [0.568–0.765]	<0.0001 ***	93.8%/38.3%/66.3%	+
rs144929717	0.0014	0.7509	0.510 [0.405–0.614]	0.7533	100%/0%/50.5%	-
rs139228330	0.0014	0.7509	0.510 [0.405–0.614]	0.7533	100%/0%/50.5%	-
Age	0.0050	0.4976	0.548 [0.443–0.807]	0.4195	39.6%/74.5%/56.8%	-
Number of all teeth erupted	0.0104	0.3882	0.563 [0.457–0.664]	0.2454	64.6%/51.1%/57.9%	-
Nr of incisors	0.0192	0.2408	0.510 [0.406–0.614]	0.3173	2.1%/100%/50.5%	-
Nr of canines	0.0019	0.7104	0.529 [0.424–0.632]	0.2803	10.4%/95.7%/52.6%	-
Nr of molars	0.0235	0.1940	0.572 [0.467–0.673]	0.1702	64.6%/51.7%/57.9%	-

Abbreviations: AUC—area under the receiver operating characteristic; 95% CI—the 95% Confidence Interval; ^1^ the column depicts variables that were statistically significant (+) or insignificant (−) for the differences in genotype/allele distribution in caries vs. caries-free individuals in the previous studies [14,15] and/or in univariable logistic regression and/or ROC curve analysis in this study. *p*-values: *p* < 0.05 *, *p* < 0.01 **, *p* < 0.001 ***; all significant results are shown in bold.

**Table 7:** The overall performance of multivariable logistic regression analysis.

Test	
**Overall model evaluation**	
Null model-2 Log Likelihood	131.69
Full model-2 Log Likelihood	42.09
Chi^2^	89.59
*df*	15
*p*-value	<0.0001 ***
Cox and Snell R^2^	0.6106
Negelkerke R^2^	0.8141
**Goodness-of-fit test**	
Hosmer and Lemeshow test:	
Chi^2^	1.58
*df*	7
*p*-value	0.9793

Abbreviations: *df*—degrees of freedom; Cox and Snell R^2^ and Negelkerke R^2^—pseudo R^2^s that indicate how well the model explains the data; for both coefficients, the closer the value is to “1”, the better the model. Hosmer and Lemeshow test—explains how well the data fit the model; the closer the value is to “1”, the better the data are fitted. *p*-values: *p* < 0.001 ***.

**Table 8:** The multivariable logistic regression analysis.

Predictor	β Coefficient	SEβ	Wald’s Chi^2^	*df*	*p*-Value	e^β^ (Odds Ratio)	[95% CI]
Intercept	0.021	0.205	0.011	1	0.918	1.021	-
rs10429371_CC	1.101	1.04	1.13	2	0.2882	3.01	[0.39–22.94]
rs10429371_TT	34.4928	13,615.64	6.42 × 10^−6^	1	0.998	9.55 × 10^14^	-
rs7096206_CG	−0.6751	1.06	0.41	1	0.523	0.51	[0.06–4.04]
rs7096206_GG	−19.4524	8976.37	4.69 × 10^−6^	1	0.9983	3.56 × 10^−9^	-
rs34538475_GT	0.1413	1.09	0.02	1	0.8969	1.15	[0.14–9.77]
rs17878486_CT	1.6945	1.09	2.41	1	0.1207	5.44	[0.64–46.28]
**rs17878486_TT**	5.4355	1.63	11.15	1	0.0008 ***	229.41	[9.45–5576.54]
**rs2337360_AA**	3.1515	1.1	8.29	2	0.0040 **	23.37	[2.73–199.80]
rs2337360_GG	19.7138	42,999.26	2.10 × 10^−7^	1	0.9996	3.64 × 10^8^	-
rs2235091_AG	1.1351	1.01	1.26	1	0.2623	3.11	[0.43–22.64]
rs2235091_GG	1.7072	1.66	1.06	1	0.3025	5.51	[0.22–141.52]
rs198969_CC	−0.2473	1.03	0.06	2	0.811	0.78	[0.10–5.93]
rs198969_GG	1.0457	1.72	0.37	1	0.542	2.85	[0.10–82.01]
rs12640848_AA	0.2135	1.08	0.04	2	0.8432	1.24	[0.15–10.26]
**rs12640848_GG**	−3.7776	1.84	4.21	1	0.0401 *	0.02	[0.001–0.84]
Constatnt	−3.9125	1.68	5.44	1	0.0197 *	-	-

Abbreviations: SNPs—single nucleotide polymorphisms; SE—standard error; *df*—degrees of freedom; 95% CI—the 95% Confidence Interval; A—adenine, C—cytosine, G—guanine, T—thymine. Intercept represents the regression constant in the model without predictors; *p*-values: *p* < 0.05 *, *p* < 0.01 **, *p* < 0.001 ***; all significant results are shown in bold.

**Table 9:** The overall performance for 6 neural network models for caries prediction.

Neural Network ID	Train		Test		Validation		Learning Algorithm ^1^	Error Rate Function ^2^	Activation Function (Hidden Layers)	Activation Function (Output Layer)
	Accuracy	Error	Accuracy	Error	Accuracy	Error				
NN1	0.970	0.007	0.984	0.004	0.736	0.084	BFGS 28	SOS	Tanh	Tanh
NN2	0.805	0.044	0.909	0.021	0.872	0.037	BFGS 5	SOS	Exponential	Exponential
NN3	0.840	0.037	0.944	0.016	0.854	0.038	BFGS 4	SOS	Linear	Logistic
NN4	0.842	0.036	0.935	0.017	0.853	0.038	BFGS 4	SOS	Tanh	Logistic
NN5	0.824	0.040	0.962	0.012	0.859	0.041	BFGS 7	SOS	Logistic	Logistic
NN6	0.867	0.031	0.912	0.022	0.862	0.038	BFGS 10	SOS	Tanh	Exponential

Abbreviations: ID—identification; NN—neural network; ^1^ The Broyden–Fletcher–Goldfarb–Shanno (BFGS) algorithm; ^2^ symbiotic organisms search (SOS) error rate.
